# Long-latency interhemispheric interactions between motor-related areas and the primary motor cortex: a dual site TMS study

**DOI:** 10.1038/s41598-017-13708-2

**Published:** 2017-11-02

**Authors:** Francesca Fiori, Emilio Chiappini, Matteo Candidi, Vincenzo Romei, Sara Borgomaneri, Alessio Avenanti

**Affiliations:** 1 IRCCS Fondazione Santa Lucia, 00179 Rome, Italy; 2 Centro studi e ricerche in Neuroscienze Cognitive, Dipartimento di Psicologia, Università di Bologna, 47521 Cesena, Italy; 3 Dipartimento di Psicologia, Sapienza Università di Roma, 00185 Roma, Italy; 4 Centre for Brain Science, Department of Psychology, University of Essex, CO4 3SQ Colchester, UK

## Abstract

The primary motor cortex (M1) is highly influenced by premotor/motor areas both within and across hemispheres. Dual site transcranial magnetic stimulation (dsTMS) has revealed interhemispheric interactions mainly at early latencies. Here, we used dsTMS to systematically investigate long-latency causal interactions between right-hemisphere motor areas and the left M1 (lM1). We stimulated lM1 using a suprathreshold test stimulus (TS) to elicit motor-evoked potentials (MEPs) in the right hand. Either a suprathreshold or a subthreshold conditioning stimulus (CS) was applied over the right M1 (rM1), the right ventral premotor cortex (rPMv), the right dorsal premotor cortex (rPMd) or the supplementary motor area (SMA) prior to the TS at various CS-TS inter-stimulus intervals (ISIs: 40–150 ms). The CS strongly affected lM1 excitability depending on ISI, CS site and intensity. Inhibitory effects were observed independently of CS intensity when conditioning PMv, rM1 and SMA at a 40-ms ISI, with larger effects after PMv conditioning. Inhibition was observed with suprathreshold PMv and rM1 conditioning at a 150-ms ISI, while site-specific, intensity-dependent facilitation was detected at an 80-ms ISI. Thus, long-latency interhemispheric interactions, likely reflecting indirect cortico-cortical/cortico-subcortical pathways, cannot be reduced to nonspecific activation across motor structures. Instead, they reflect intensity-dependent, connection- and time-specific mechanisms.

## Introduction

Motor network functioning is based on neural interactions between different premotor and motor areas. The frontal lobe contains multiple premotor areas that are involved in action planning and execution and in a number of motor and cognitive processes including motor imagery^1–3^, action perception^4,5^ and language production and comprehension^6,7^. Premotor areas are known to act in concert with the primary motor cortex (M1) during motor behavior and, interestingly, part of this interplay occurs via interhemispheric interactions^8–10^. Neuroimaging studies have revealed high functional coupling between activity in premotor regions and the contralateral M1 even when people are at rest^11–13^. However, these studies rely on a correlational approach characterized by low temporal resolution^14,15^. Neurophysiological techniques appear better suited for disclosing the time-course of premotor-M1 causal interactions. Yet, how premotor and motor areas in one hemisphere causally interact with the contralateral M1 is still poorly understood.

Evidence of premotor-motor interhemispheric interactions can be gathered using the dual-site transcranial magnetic stimulation (dsTMS) protocol^16–22^. In the dsTMS paradigm, a suprathreshold test stimulus (TS) administered to M1 is preceded by a conditioning stimulus (CS) administered to an interconnected brain region (e.g., in the contralateral hemisphere) at a selected inter-stimulus interval (ISI). The CS activates hypothetical pathways (employing direct/indirect connections) from the conditioning site to M1 and modulates the amplitude of motor-evoked potentials (MEPs) elicited by the TS. Depending on CS intensity, location, and the ISI between the CS and the TS, both facilitatory and inhibitory influences on M1 activity can be detected^23^, thus providing causal physiological evidence for the directionality and timing of cortico-cortical interactions.

Seminal dsTMS studies have reported that M1 stimulation in one hemisphere inhibits the excitability of the contralateral M1^16,17,19,20^. This effect takes place via transcallosal pathways and is referred to as interhemispheric inhibition. A large body of studies reported short-latency interhemispheric interactions (ISIs < 15 ms) between the left and right M1^16,17,19,20,24–26^. However, longer-latency interactions between the two M1 areas have also been documented^19,21,22^. Those interactions are altered in neurological conditions affecting motor control^27,28^, suggesting that motor network functioning might hinge on the optimal tuning between short- and long-latency interhemispheric interactions.

More recently, dsTMS has been employed to investigate connectivity between non-homologous areas, i.e., between premotor areas in one hemisphere and the contralateral M1. Studies have documented that M1 excitability can be affected not only by conditioning the contralateral M1 but also by a CS administered over the contralateral dorsal premotor cortex (PMd)^8,29–31^ or the ventral premotor cortex (PMv)^32–34^. Moreover, studies have tested the influence of a CS over the supplementary motor area (SMA) on M1 excitability^33,35–37^ (it should be noted that in this case the CS likely affects SMA bilaterally, and thus the modulatory effects on M1 may also reflect the influence of the ipsilateral SMA).

Importantly, all these studies have focused on short ISIs only (typically < 20 ms), while investigations of long-lasting interactions have been mainly limited to homologous M1 areas only. Studies of long-latency interactions between premotor areas and the contralateral M1 are scarce. To the best of our knowledge, only two studies have investigated such interactions. Ni *et al*.^22^ tested the influence of right M1 (rM1) and right PMd (rPMd) conditioning on the excitability of the left M1 (lM1). Mochizuki *et al*.^21^ investigated the influence of a CS administered over right motor-related areas (rM1 and a dorso-lateral premotor site at the border between rPMd and the right PMv, rPMv) on the excitability of lM1. These studies have documented longer-latency premotor-motor interhemispheric interactions, supporting the notion that motor network functioning might rely on interactions at different time-scales. However, they did not clarify the issue of anatomical specificity, i.e., whether different sectors of premotor cortex (i.e., from ventral to medial areas) exert different effects on contralateral M1 excitability. Notably, these studies reported very similar modulatory effects when testing interhemispheric interactions between non-homologous areas (i.e., when a CS was administered over rPMd and a TS over lM1) and when testing motor-motor interhemispheric interactions (i.e., a CS over rM1 and a TS over lM1). This raises the possible concern that, at long ISIs, causal interactions from premotor/motor sites to contralateral M1 may reflect a nonspecific spreading of activation across motor structures. Indeed, long-latency interactions likely reflect complex and indirect pathways^19,33^. However, the apparently nonspecific interhemispheric effects reported in the two previous studies of Mochizuki *et al*.^21^ and Ni *et al*.^22^ could be partly due to the high suprathreshold CS intensities used. Indeed, in those studies, lower (i.e., subthreshold) CS intensities were only used at a single long-latency ISI of 50 ms^22^, but not at later ISIs.

In a third, recent study by Fiori *et al*.^18^, our group also tested long-latency interactions between a rostral medial premotor site and lM1. Although we found site-specific effects of medial premotor subthreshold and suprathreshold conditioning over M1, our study did not focus on interhemispheric interactions, and thus did not include rM1 as a control CS site.

Therefore, an important and yet unanswered question is to what extent distinct ventral, dorsal and medial sectors of the premotor cortex in one hemisphere exert site-specific modulatory effects over the contralateral M1 resulting in a long-latency influence that is distinct from the influence exerted by M1 over its contralateral homologue. Disclosing site-specific premotor-motor interactions requires a systematic investigation of the effect of the CS location, but also CS intensity, as different TMS intensities can recruit partially distinct neural populations^18,38^.

All these issues are addressed in the present study, which investigated how CS intensity and CS location within different premotor and motor areas in one hemisphere impacted the excitability of the contralateral M1 at long ISIs. To this aim, we used a dsTMS protocol while recording MEPs at rest. To compare our data with those of Ni *et al*.^22^, Mochizuki *et al*.^21^ and Fiori *et al*.^18^, we focused on the influence that a CS over right hemispheric motor areas exerts over the contralateral M1. Therefore, the TS was administered over lM1, and MEPs were recorded from the right hand. The TS was either administered alone (single pulse TMS) or preceded by a CS over one of four sites: rM1, rPMv, rPMd and the SMA (for technical reasons, the SMA was stimulated bilaterally, as in previous research^33,35–37^). To explore long-latency interactions, the ISI between the CS and the TS was varied between six time intervals (40, 60, 80, 100, 120 and 150 ms). Furthermore, to test the effect of CS intensity, we administered either a subthreshold CS (i.e., 90% of the resting motor threshold, rMT) or a suprathreshold CS (i.e., 110% of rMT). This experimental design allowed us to track the time course and the CS-intensity dependence of interhemispheric premotor-motor interactions. Our study shows that different sectors of the premotor cortex exert site-specific modulatory influences over the contralateral M1. Moreover, our study highlights, for the first time, the strong modulatory influence exerted by rPMv over lM1. Our findings suggest that long-latency PMv-M1 interhemispheric interactions may be a novel, powerful target for modulating motor network functioning in both healthy and damaged brains^39,40^.

## Methods

### Participants

Fifteen right-handed healthy participants (6 males; mean age ± S.D.: 25.2 ± 2.3 years) took part in this study. All participants gave their informed written consent before being tested. The experimental procedures were approved by the University of Bologna Bioethics committee and were in accordance with the 1964 Declaration of Helsinki. The methods carried out in this study are in accordance with approved guidelines. No adverse reactions to TMS were noticed during stimulation or reported by participants^41^.

### Procedure

Participants underwent 8 blocks of stimulation, following a 4 (CS site: rPMv, rPMd, SMA or rM1) × 2 (CS intensity: 90% and 110% of the rMT) blocked factorial design. Additionally, in each block, the TS was either administered alone (single pulse TMS: spTMS) or coupled with a preceding CS (dsTMS) delivered at one of 6 ISIs (40, 60, 80, 100, 120 or 150 ms). The order of the blocks and the TMS conditions (spTMS/dsTMS at various ISIs) within each block were randomized. Each block consisted of 152 trials (120 dsTMS trials, 20 at each ISI, and 32 spTMS trials) with a fixed inter-trial interval of 6 s. The block was split into 2 parts (with a short break in between) and lasted about 18 minutes. A 5 min break was allowed between blocks. Due to the overall duration of the experiment, testing was divided into two sessions conducted on two different days (4 blocks per day), separated by 7 ± 3 days. Participants sat on a comfortable chair. They were asked to shut their eyes and keep both hands relaxed while testing, with the aim of obtaining a stable electromyographic (EMG) signal and minimizing any visual distractions.

### Electromyography and TMS

Silver/silver chloride electrodes were placed in a belly-tendon montage on the right first dorsal interosseous (FDI) muscle. EMG signals from the FDI were recorded by means of a Biopac MP-35 (Biopac, USA) electromyograph, using a band-pass filter of 30–500 Hz and a sampling rate of 5000 Hz. TMS pulses were administered via two 50-mm butterfly-shaped coils, each of which was connected to a Magstim 200 monophasic transcranial stimulator (Magstim, UK).

The TS was administered over lM1 with the intersection of the coil placed tangentially to the scalp, at a ~45° angle away from the midline, inducing a posterior-to-anterior current direction^42,43^. The lM1 was identified as the optimal scalp position for inducing the largest MEPs in the right FDI. The TS intensity was set in order to induce MEPs of ~1 mV amplitude. The corresponding mean stimulator output ± S.D. was 53.4% ± 11.5 on day 1 and 52.6% ± 13.0 on day 2 (P = 0.49). The CS was administered over rM1 (corresponding to the hotspot for evoking the largest MEPs in the left FDI), and over rPMv, rPMd and SMA, all of which were localized using established methods (see next paragraph). The CS intensity was either subthreshold or suprathreshold, corresponding to 90% and 110% of the rMT, respectively. The rMT was defined as the lowest stimulator intensity able to evoke a MEP larger than 50 µV with 50% probability. The mean rMT ± S.D. across participants was 40.3% ± 6.5 on day 1 and 41.4% ± 8.0 on day 2.

### Stimulation sites

To localize the stimulation sites, we used established functional, craniometric and stereotaxic procedures. Each target site was identified on the scalp based on the most established procedure (e.g., functional methods for M1), and then the position of the coil was verified using a neuronavigation system^18^. Both the lM1 and the rM1 scalp sites were localized using functional procedures, i.e., by identifying the FDI motor hotspot. The rPMd scalp site was determined by placing the coil 2.5 cm anterior and 1 cm medial relative to rM1 as in previous research^22,31,44^. For this stimulation site, the TMS coil was rotated away from the sagittal midline by ~ 90°, inducing a lateral-to-medial current^21,22^. When stimulating the SMA, the coil was positioned 4 cm anterior to the Cz position in the 10–20 system^35,45–47^, and the handle of the coil was pointed forward to induce an anterior-to-posterior current^35,46^.

The rPMv scalp site was identified using a neuronavigation system^18,48–50^. We used the SofTaxic Navigator system (EMS; Electro Medical Systems, Bologna, Italy), as in previous studies^18,51–55^. This system automatically estimates Talairach coordinates from a magnetic resonance imaging (MRI)-constructed stereotaxic template. Based on the MRI template, we estimated the scalp position corresponding to rPMv (on the anterior ventral aspect of the precentral gyrus, at the border with the posterior part of the inferior frontal gyrus) using the Talairach coordinates x = 52, y = 7, z = 24. The center of the coil was positioned over this location with the handle pointing anteriorly, inducing a posterior-to-anterior current^49,56–58^.

**Figure 1:**
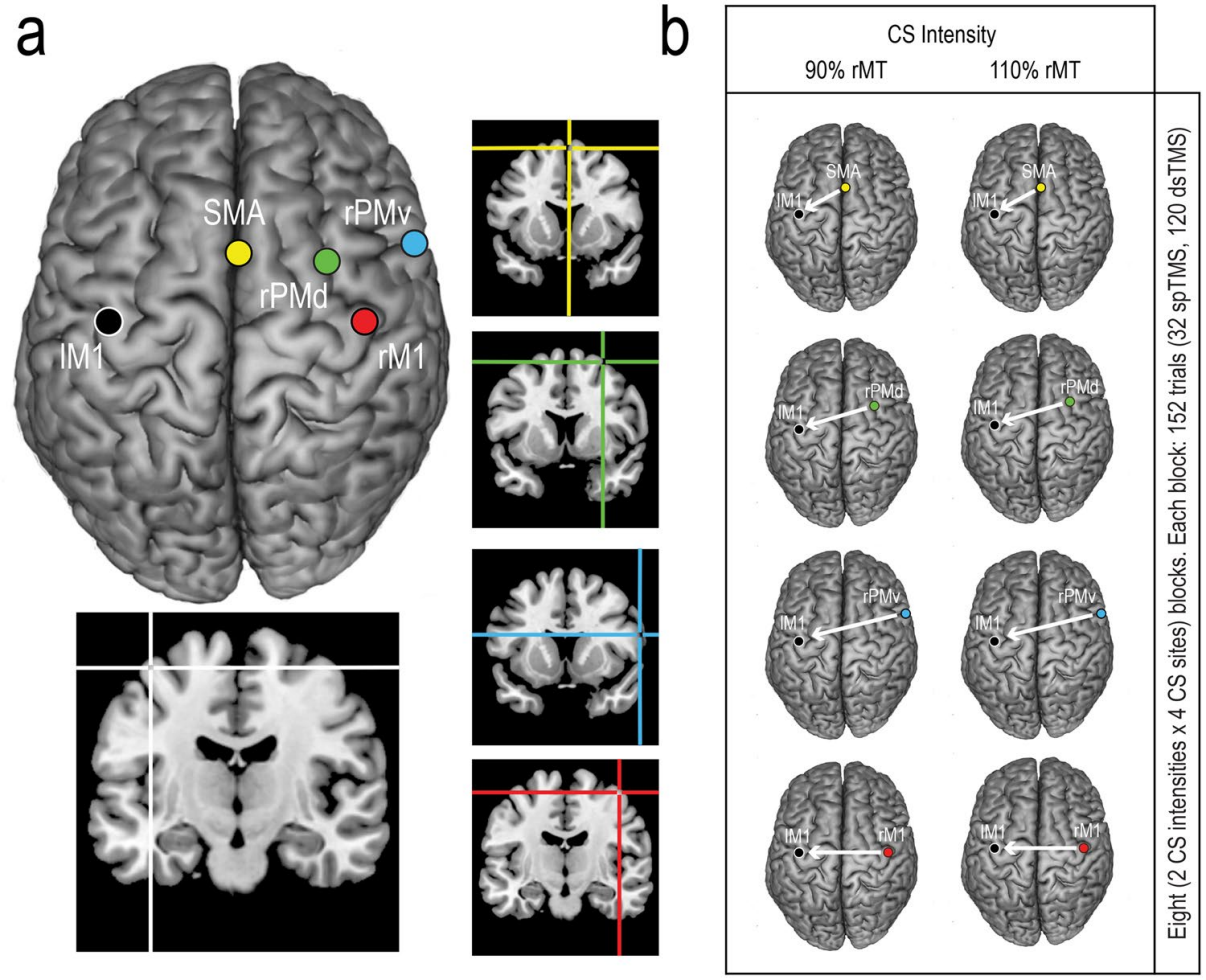
**(a)** Brain stimulation sites. Coordinates in Talairach space corresponding to the projection of the stimulated scalp sites onto the brain surface were estimated through a neuronavigation system and reconstructed on a standard template using MRIcron software (v 1.40, ww.mricro.com). (**b**) Schematic representation of the experimental blocks. For each experimental block, brain stimulation sites, CS intensity and number of trials are reported.

The neuronavigation system was also used to estimate the coordinates of the target locations (lM1, rM1, rPMv, rPMd and SMA) projected onto the cortical surface of the MRI template (see Fig. 1). For each participant, skull landmarks (nasion, inion and 2 preauricular points) and about 80–100 points providing a model of the scalp were digitized through a Polaris Vicra digitizer (Northern Digital). An estimated MRI was created for each participant using a 3D warping algorithm that fits a high-resolution MRI template to the acquired landmarks and scalp model. This estimation has been proven to ensure a spatial accuracy of ~5 mm, a level of precision closer to that obtained using individual MRIs than can be achieved using other localization methods^59^. The mean ± S.E.M. estimated Talairach coordinates were: lM1: x = −39.3 ± 1.0, y = −19.0 ± 1.6, z = 58.6 ± 0.9; rM1: x = 37.6 ± 1.2, y = −18.5 ± 1.8, z = 58.6 ± 1.1; rPMv: x = 54.3 ± 0.9, y = 7.3 ± 0.5, z = 23.4 ± 0.4; rPMd: x = 25.8 ± 2.0, y = 1.0 ± 2.2, z = 62.9 ± 1.6; and SMA: x = 0.6 ± 0.2, y = 4.9 ± 1.8, z = 63.8 ± 1.6. These estimated coordinates are consistent with the boundaries of human M1, PMv, PMd and SMA regions as defined by a meta-analysis of neuroimaging studies^60^. They are also consistent with previous TMS studies that used individual’s MRI data to localize these areas for stimulation^9,31,42,45–47,61–64^.

### Data analysis

Neurophysiological data were analyzed offline. Due to a technical issue, data from one female participant were lost, so the final sample consisted of fourteen individuals. EMG activity was visually inspected, and trials showing muscle activity 100 ms before the TMS artifact were removed from the analysis (~4%). In each block, the mean peak-to-peak MEP amplitude was calculated for dsTMS and spTMS conditions. In each condition, MEPs with an amplitude ≥ 2 S.D. from the mean were excluded from the analysis (~3% of trials).

A repeated measures ANOVA with the factors CS site (4 levels: rPMv, rPMd, rM1 and SMA) and CS intensity (2 levels: 90% and 110% rMT) was first conducted on raw MEP amplitudes induced by spTMS (TS alone). Neither of the main effects was significant, nor was the interaction (all P > 0.26), demonstrating that MEPs induced by spTMS were comparable across the eight blocks. Then, MEPs elicited by spTMS were used to normalize the MEP amplitudes induced by dsTMS: in each block (i.e., for each combination of CS site and CS intensity), an index of dsTMS modulation was computed for each ISI by subtracting MEPs elicited by spTMS within the same block from MEPs elicited by dsTMS (dsTMS MEP – spTMS MEP). Normalized dsTMS modulation indices were submitted to a repeated measures ANOVA with the factors CS site (4 levels: rPMv, rPMd, rM1 and SMA), CS intensity (2 levels: 90% and 110% of rMT) and ISI (6 levels: 40, 60, 80, 100, 120 and 150 ms). Partial η^2^ (η_p_^2^) was computed as a measure of effect size for significant main effects and interactions. By convention, η_p_^2^ effect sizes of ~0.01, ~0.06, and ~0.14 are considered small, medium, and large, respectively^65^.

The ANOVA showed a significant three-way interaction (see Results section) which was further explored with six separate CS site x CS intensity ANOVAs, one for each ISI. In these further ANOVAs, we directly tested the critical question of whether rPMv, rPMd or SMA exert site-specific modulatory influences over lM1 that differ from the modulatory influence exerted by rM1. We used post-hoc pairwise comparisons (Duncan’s tests) to analyze significant effects involving the factor CS site. Additionally, to better interpret the pattern of results shown in the ANOVAs, we used one-sample t-tests to test whether dsTMS modulation indices (dsTMS MEP – spTMS MEP) differed significantly from zero (i.e., whether MEPs in the dsTMS conditions were different from the corresponding spTMS condition).

## Results

**Figure 2:**
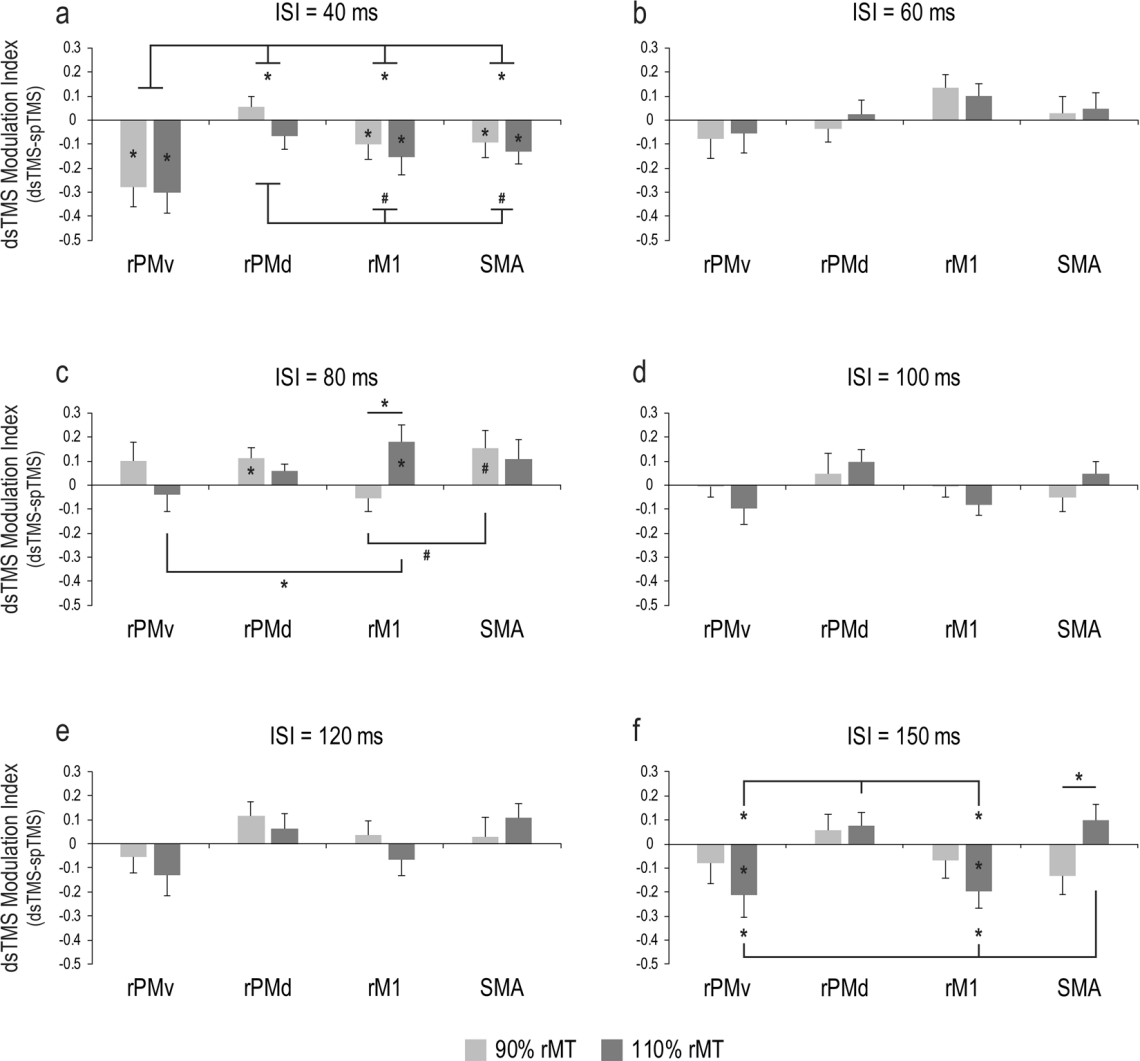
Changes in lM1 excitability induced by conditioning of right motor areas. The CS site (rPMv, rPMd, rM1 and SMA) x CS intensity (90% and 110% of rMT) interaction is shown separately for each ISI (40, 60, 80, 100, 120, 150 ms) in panels (**a–f**). On the y-axis of each panel, the amplitude of MEPs induced by dsTMS is represented relative to MEPs induced by spTMS (dsTMS – spTMS) to normalize the data. Error bars denote S.E.M. Hash marks and asterisks indicate marginally significant and significant comparisons, respectively (see text).

The CS site x CS intensity x ISI ANOVA on dsTMS MEP indices (i.e., dsTMS MEP – spTMS MEP) showed a main effect of CS site (F_3,39_ = 4.58, P = 0.008; η_p_^2^ = 0.26), a main effect of ISI (F_5,65_ = 7.92, P = 0.00001; η_p_^2^ = 0.38), a CS site x ISI interaction (F_15,195_ = 1.90, P = 0.025; η_p_^2^ = 0.13) and a three-way CS site x CS intensity x ISI interaction (F_15,195_ = 1.89, P = 0.026; η_p_^2^ = 0.13). The three-way interaction indicates that the combined influence of CS intensity and ISI on MEP amplitudes varied as a function of the CS site, thus providing initial support to the hypothesis of site-specific effects. To further explore the three-way interaction and test site-specific modulatory influences of premotor/motor conditioning on lM1 excitability, separate CS site x CS intensity ANOVAs were performed, one for each ISI (Fig. 2; see also Supplementary Fig. S1).

### 40-ms ISI

The CS site x CS intensity ANOVA performed on the dsTMS modulation index collected at a 40-ms ISI showed a main effect of CS site (F_3_,_39_ = 7.26, P = 0.001; η_p_^2^ = 0.36; Fig. 2a). Post-hoc comparisons suggested that this main effect was accounted for by the more negative dsTMS modulation index values obtained with rPMv conditioning (mean MEP contrast ± S.E.M. = −0.29 mV ± 0.08) relative to rPMd (−0.01 mV ± 0.02; P = 0.001), rM1 (−0.13 mV ± 0.05; P = 0.012) and SMA conditioning (−0.11 mV ± 0.04; P = 0.009). This result reflects greater inhibition of MEPs due to rPMv conditioning, compared to the other conditioning sites. Moreover, the dsTMS modulation indices were comparable in the rM1 and SMA conditions (P = 0.81), and more negative in those conditions than in the rPMd condition (rM1: P = 0.065; SMA: P = 0.086). The ANOVA did not show a main effect of CS intensity or an interaction between the two factors (F < 1.70, P > 0.21), suggesting that the site-specific modulations at a 40-ms ISI were not affected by CS intensity.

One-sample t-tests were performed to further explore the main effect of CS site. These analyses showed that the dsTMS modulation index (across the two CS intensities) was significantly less than zero (i.e., dsTMS MEPs were inhibited relative to spTMS MEPs) in the rPMv, rM1 and SMA conditions (all P < 0.027), but not in the rPMd condition (P = 0.78). Thus, conditioning the rPMv, rM1 and SMA with dsTMS elicited motor inhibition relative to (unconditioned) spTMS MEPs.

### 60-ms ISI

At this ISI, no significant effects were detected (Fig. 2b). The CS site x CS intensity ANOVA showed no significant main effect of CS site (F_3_,_39_ = 2.47, P = 0.076; η_p_^2^ = 0.16) and no main effect of, or interaction with, CS intensity (F < 0.56, P > 0.65).

### 80-ms ISI

This ANOVA showed a significant CS site x CS intensity interaction (F_3_,_39 = _3.20, P = 0.034; η_p_^2^ = 0.20; Fig. 2c), but no significant main effects (all F < 0.97, all P > 0.54). The interaction was due to the different influences exerted by subthreshold and suprathreshold CS intensities across CS sites. Post-hoc analyses showed that when the CS was administered over rM1, more positive dsTMS modulation index values were obtained with a suprathreshold CS compared to a subthreshold CS (0.18 mV ± 0.07 vs. −0.06 mV ± 0.05; P = 0.031). An opposite pattern of rPMv, rPMd and SMA conditioning was appreciable by visual inspection (i.e., more positive dsTMS modulation indices for a subthreshold CS than for a suprathreshold CS), but the relevant post-hoc tests did not reach statistical significance (all P > 0.15). The CS site x CS intensity interaction was also due to larger (more positive) dsTMS modulation indices with suprathreshold rM1 conditioning than with suprathreshold rPMv conditioning (P = 0.042). Also, a larger dsTMS modulation index was found with subthreshold conditioning when the CS was delivered to the SMA than when it was administered over rM1 (P = 0.051). No other comparisons were significant (all P > 0.49).

One-sample t-tests were used to further explore the significant interaction. These tests showed that dsTMS modulation indices were significantly greater than zero (i.e., dsTMS MEPs were facilitated relative to spTMS MEPs) when using a suprathreshold CS over rM1 (P = 0.025), and a subthreshold CS over rPMd (P = 0.019) and the SMA (P = 0.056). Facilitation with a subthreshold CS over the SMA was marginally significant (P = 0.056), and facilitation with a subthreshold CS over rPMv did not reach significance (P = 0.21). No other conditions showed dsTMS modulation indices different from zero (all P > 0.22).

### 100- and 120-ms ISIs

At these ISIs, no significant effects were detected (Fig. 2d,e). The CS site x CS intensity ANOVAs showed no significant main effects of CS site at 100 ms (F_3_,_39_ = 2.31, P = 0.092; η_p_^2^ = 0.15) or 120 ms (F_3_,_39_ = 2.45, P = 0.08; η_p_^2^ = 0.16) and no main effects of, or interactions with, CS intensity (all F < 1.10, all P > 0.36).

### 150-ms ISI

The ANOVA showed a main effect of CS site (F_3_,_39_ = 3.46, P = 0.026; η_p_^2^ = 0.21), but no main effect of CS intensity (F < 0.01, P = 0.96). It also showed a significant CS site x CS intensity interaction (F_3_,_39_ = 3.63, P = 0.021; η_p_^2^ = 0.22; Fig. 2f). Post-hoc analyses showed more negative dsTMS modulation indices when suprathreshold conditioning was administered over rPMv (−0.21 mV ± 0.09) and rM1 (−0.20 mV ± 0.07) relative to rPMd (0.08 mV ± 0.05; all P < 0.01) and SMA (0.10 mV ± 0.07; all P < 0.006), which in turn did not differ from one another (P = 0.8). Suprathreshold conditioning of rPMv and rM1 induced comparable dsTMS modulation indices (P = 0.84). One-sample t-tests indicated that rPMv and rM1 dsTMS modulation indices were significantly different from zero (i.e., dsTMS MEPs were inhibited relative to spTMS MEPs; all P < 0.037). No other conditions showed dsTMS modulation indices different from zero (all P > 0.16).

Post-hoc analyses also showed that the comparison between suprathreshold and subthreshold CS intensities was significant when the CS was administered over the SMA (P = 0.028), but not when the CS was administered over rPMv, rM1 or rPMd (all P > 0.19). When the CS was administered over the SMA, dsTMS modulation indices were negative for a subthreshold CS and positive for a suprathreshold CS. However, those dsTMS modulation indices did not significantly differ from zero, as shown by one-sample t-tests (all P > 0.16).

## Discussion

Motor network functioning might depend on the optimal tuning of neural interactions between different nodes of the network. These interplays include interhemispheric interactions between homologous^17,19,66^ and non-homologous areas^30,34^. The functional interactions between these interconnected regions of the motor network likely occur at different time scales, and can be optimally explored using causal methods with high temporal resolution like the dsTMS protocol.

As reported in the introduction, the only two previous dsTMS studies demonstrating the existence of long-latency premotor-motor interhemispheric interactions in healthy humans^21,22^ showed that the effects of stimulating non-homologous areas (i.e., PMd-M1) were very similar to the effects of stimulating homologous areas (i.e., M1-M1), thus leaving unresolved the issue of the anatomical specificity of long-latency interhemispheric interactions in the human motor system. We hypothesized that the apparent lack of specificity reported in previous studies might stem from the limited number of conditions being tested. Thus, in the present study, we provided a systematic investigation of long-latency interactions (ISIs from 40 to 150 ms) between primary and non-primary motor areas of the right hemisphere (rM1, rPMv, rPMd and bilateral SMA) and lM1. We investigated the effects of ISI, CS site and CS intensity (subthreshold vs. suprathreshold intensity) on lM1 excitability, while participants were at rest.

Our study highlights three key time points (i.e., 40-, 80- and 150-ms ISIs) at which site-specific MEP modulations occurred. The first inhibitory modulation of lM1 was detected when the CS was administered over rPMv, rM1 and the SMA at a 40-ms ISI. The 80-ms ISI revealed an intensity-dependent excitatory influence of rM1 conditioning, while the 150-ms ISI highlighted intensity-dependent inhibitory influences of rPMv and rM1.

In keeping with previous studies testing long-latency cortico-cortical interactions^18,21,22^, most of the interactions detected across the CS sites and ISIs were inhibitory. Monkey studies indicate that interhemispheric interactions occur mainly through transcallosal pathways connecting homologous areas in the two hemispheres^67,68^. Transcallosal connections are constituted by excitatory fibers originating from a target (motor) area in one hemisphere (i.e., where the CS is administered) and synapsing over interneurons in the contralateral homologue. Excitatory signals conveyed by transcallosal connections activate local circuits in the homologous area that are mainly characterized by GABAergic neurons^69^, consequently resulting in a net reduction of motor output. However, in view of the long ISIs we explored in this study, it is also very likely that complex and indirect cortico-subcortical pathways might have been involved in the observed interhemispheric inhibitions (at ISIs of 40 and 150 ms), as well as interhemispheric facilitations (at an ISI of 80 ms). Yet, our study clearly demonstrates that MEP modulations are site- and intensity-specific, even at long ISIs.

A major point of novelty of our study is the investigation of long-latency interhemispheric PMv-M1 interactions. Indeed, previous studies testing long-latency interhemispheric interactions mainly focused on M1-M1 or PMd-M1. In a previous study, Mochizuki *et al*.^21^ conditioned a dorsolateral premotor site (2 cm anterior to M1), and thus might have influenced the most dorsal aspects of PMv, whereas here we centered the CS over an anterior sector of the PMv proper. Conditioning rPMv resulted in a strong modulatory influence over lM1 in the explored time window. This modulatory influence was particularly conspicuous in the first critical ISI (40 ms). This ISI was characterized by a strong inhibitory influence of rPMv conditioning on lM1 excitability. Inhibition was greater when the CS was administered over rPMv relative to the other CS sites. A reduction in lM1 excitability was also detected with rM1 and SMA conditioning, replicating previous findings of a peak in interhemispheric inhibition when a CS was administered at a 40-ms ISI over similar sites^18,19,22^. Varying CS intensity produced no substantial differences in lM1 excitability when the CS was administered to rPMv, rM1 or the SMA. Additionally, no lM1 modulation was elicited by either subthreshold or suprathreshold conditioning of rPMd. The lack of lM1 modulation when the CS was administered over rPMd is in keeping with previous data^18^, and rules out the possibility that lM1 suppression with rPMv, rM1 or SMA conditioning might be due to nonspecific factors such as the coil click^70^. In summary, data collected across the four CS sites with a 40-ms ISI provide strong support for our hypothesis of site-specific interhemispheric interactions between motor-related areas, and suggest these interactions are relatively insensitive to the intensity of the CS. Yet, it should be acknowledged that we only tested two CS intensities, both near to rMT. Thus, future studies might use lower (< 90% rMT) or higher (>110% rMT) CS intensities in order to further test intensity-dependent modulations at this ISI.

The marked modulatory influence elicited by PMv conditioning appears in line with studies using different TMS protocols and reporting strong effects of premotor conditioning on M1. Studies have shown that administering low-frequency repetitive TMS (rTMS) over ventral and lateral premotor sites can lead to stronger modulation of M1 than administering rTMS over M1 itself^4,58,71,72^, and can affect a more widespread fronto-parietal network^73^. In the dsTMS study of Fiori *et al*.^18^, conditioning the posterior inferior frontal cortex –at the border with the PMv– led to stronger (ipsilateral) M1 modulations than conditioning a medial premotor site (pre-SMA) did, and this stronger modulation was observed at several long-latency ISIs. Picazio *et al*.^34^ used dsTMS to test short-latency premotor-motor interactions. The authors reported that conditioning a right inferior frontal site – partially overlapping with our PMv site –exerted a stronger modulatory influence over lM1 than conditioning a control site (i.e., the pre-SMA). Taken together, these findings suggest that PMv sites can exert strong modulatory influences over M1. Our findings build upon previous evidence by showing that rPMv conditioning inhibits the contralateral M1 at 40 ms after the CS, and this inhibition is even larger than that induced by conditioning the homologous M1.

In monkeys, direct (heterotopic) connections between premotor cortices and the contralateral M1 have been demonstrated^67,68^, although they are believed to play a minor role in motor functioning, with most neural interactions occurring between homologous areas. Thus, the effects exerted by rPMv stimulation over lM1 could be mainly ascribed to the recruitment of indirect pathways linking the two areas. Because of the stronger effects of rPMv relative to rM1 conditioning at the 40-ms ISI, the rPMv-lPMv-lM1 pathway appears more plausible than the rPMv-rM1-lM1 pathway, although, in view of the long ISI, even more indirect cortico-subcortical pathways could be hypothesized^33^.

An effect of rPMv conditioning was also observed at the longest ISI of 150 ms, although in this case the modulation was not specific to rPMv. In keeping with the study of Mochizuki *et al*.^21^ (that conditioned M1 and a premotor site more dorsal and posterior than our PMv site), we found that suprathreshold CS intensities administered over rPMv or rM1 led to reductions in contralateral lM1 excitability. Our data expand on previous evidence by showing that the inhibitory effects were specific to suprathreshold conditioning of rPMv and rM1, as they were not found with rPMd or SMA conditioning, or with subthreshold CS intensities. Thus, our study suggests that the second long-latency peak of inhibition found at a 150-ms ISI might reflect site-specific interactions involving homologous (rM1-lM1) as well as non-homologous areas (rPMv-lM1). Our data allow us to firmly rule out the possibility that interhemispheric inhibition at an ISI of 150 ms reflects nonspecific spreading of activation to any premotor site. Yet, future studies will need to test the possibility that spreading activation across rM1 and rPMv specifically accounts for the suppression of lM1 excitability at this ISI.

In addition to inhibitory interhemispheric interactions, we also found some evidence of facilitatory interhemispheric interactions. Motor facilitations were selectively detected at an ISI of 80 ms. Greater dsTMS MEP modulation indices were obtained with rM1 conditioning when using a suprathreshold CS relative to a subthreshold CS. Suprathreshold rM1 conditioning also increased lM1 excitability relative to spTMS. An opposite pattern of modulation across the other premotor CS sites was detectable by visual inspection (i.e., larger dsTMS MEPs induced by subthreshold relative to suprathreshold CS). Yet, subthreshold conditioning of rPMd and SMA significantly increased lM1 excitability relative to spTMS.

Previous studies have already documented short-latency M1 facilitation when the CS was administered over the contralateral M1, rPMd or the SMA. These effects were detected with both subthreshold and suprathreshold CS intensities, although not always in a consistent way^17,20,29,35^. The mechanism underlying such short-latency interhemispheric interactions is likely different from that underlying our long-latency modulations. However, it is interesting to note that these previous investigations concluded that premotor-M1 interactions and M1-M1 interactions were mediated by different populations of neurons in M1, suggesting site-specific mechanisms.

Our data appear to be in keeping with previous evidence that rPMd conditioning requires subthreshold intensities to produce interhemispheric facilitation in the contralateral M1^29,37^, and suggest that this rule may apply to long-latency interactions at an ISI of about 80 ms. On the other hand, different rules might apply to short- and long-latency interactions involving rM1 and SMA. At an 80-ms ISI, our data are not consistent with evidence that subthreshold rM1 conditioning^29^ and suprathreshold, but not subthreshold, SMA conditioning^35^ induce short-latency M1 facilitation. Yet, in those studies, the intensity of subthreshold conditioning (60–90% of active motor threshold) was much lower than that used in the present study, and the intensity of suprathreshold conditioning (140% of active motor threshold) was higher.

The selectivity of MEP facilitation for suprathreshold rM1 conditioning likely reflects site-specific and intensity-dependent interactions between the two homologous M1 areas. A possible alternative interpretation is that suprathreshold conditioning of rM1 may have caused a spreading of the magnetic stimulation to nearby premotor CS sites (e.g., rPMd or SMA), resulting in attenuated activation of those sites, similar to that caused by subthreshold CS intensities over the same sites. However, the M1-M1 facilitatory effect with suprathreshold conditioning was more consistent than the premotor-M1 facilitatory effects with subthreshold conditioning, thus speaking in favor of site-specific interactions between the two M1 areas. Nevertheless, future studies will have to clarify whether the same circuit mediates the effects observed with rM1 and premotor conditioning at an ISI of 80 ms.

A potential limitation of our study is the use of only two levels of CS intensity. The investigation of an input-output curve with additional CS intensities (i.e., lower subthreshold and higher suprathreshold intensities) may further highlight intensity-dependent interhemispheric interactions at specific ISIs. Moreover, based on the previous studies of Ni *et al*.^22^ and Mochizuki *et al*.^21^, we focused on right-to-left interhemispheric interactions in right-handed participants only. In this regard, future studies might test whether asymmetrical interactions occur at late ISIs, similar to those reported with short ISIs^8,30,74^. Yet, the selected CS intensities and the focus on right-to-left cortico-cortical interactions were sufficient to support our hypothesis that long-latency interhemispheric interactions cannot be reduced to a nonspecific spreading of activation across motor structures.

In conclusion, our study documents site- and intensity-dependent inhibitory and facilitatory modulations of lM1 excitability by stimulation of contralateral premotor and motor regions in the right hemisphere. Our data highlight prominent and distinct modulatory roles of rPMv, rM1 and SMA over lM1 across the explored ISIs of 40–150 ms. Although the reported modulations at 40-, 80- and 150-ms ISIs likely reflect not only the recruitment of direct pathways but also large indirect cortico-cortical and cortico-subcortical pathways^15,19,33,75^, our study clarifies that long-latency interhemispheric interactions do not reflect a nonspecific spreading of activation across motor structures^21,22^. Rather, they reflect intensity-dependent, site- and time-specific mechanisms.

The investigation of long-latency interhemispheric interactions is important for understanding the rules governing motor network functioning at rest, and can lay the groundwork for further exploration during motor and/or cognitive tasks that involve premotor-to-motor connectivity^61,76–78^ or connections between other sectors of the motor system^79–81^. Tracking the specific time course of interhemispheric interactions between homologous and non-homologous brain areas in the healthy population can provide novel insights into clinical conditions associated with altered connectivity patterns. Our study does not clarify which pathways mediate these neurophysiological interactions. Nevertheless, our findings point to specific time intervals at which motor and premotor areas can affect contralateral M1 output. Studies of the exact time scales of these interactions are of potential interest, as they might be crucial for manipulating the functionality of these motor connections. For example, one might apply novel TMS protocols such as the cortico-cortical paired associative stimulation (cc-PAS), which can modify the functional connectivity between interconnected nodes^63,82–84^. Future applications of these kinds of non-invasive neurostimulation protocols are promising for clinical profiles characterized by altered connectivity across functional networks^39,66^.

## Electronic supplementary material

Supplementary Information
