# Supplementary material for: Long-latency interhemispheric interactions between motor-related areas and the primary motor cortex: a dual site TMS study
Source: Sci Rep. 2017 Nov 2;7:14936. doi: 10.1038/s41598-017-13708-2 (PMC5668244; doi:10.1038/s41598-017-13708-2)
Supplement: Supplementary file 1 — Supplementary Information [file 41598_2017_13708_MOESM1_ESM.pdf]

Long-latency interhemispheric interactions between motor-related areas and the primary motor cortex: a dual site TMS study

Fiori F[1,2,3*], Chiappini E[1,2*], Candidi M[1,3], Romei V[4], Borgomaneri S[1,2], Avenanti A[1,2]

[1] IRCCS Fondazione Santa Lucia, 00179 Rome, Italy;

[2] Dipartimento di Psicologia, Università di Bologna and Centro studi e ricerche in Neuroscienze Cognitive, Campus di Cesena, Università di Bologna 47521 Cesena, Italy;

[3] Dipartimento di Psicologia. Sapienza Università di Roma;

[4] Centre for Brain Science, Department of Psychology, University of Essex, UK.

[*] These authors equally contributed to this work

Corresponding author:

Alessio Avenanti. E-mail: alessio.avenanti@unibo.it

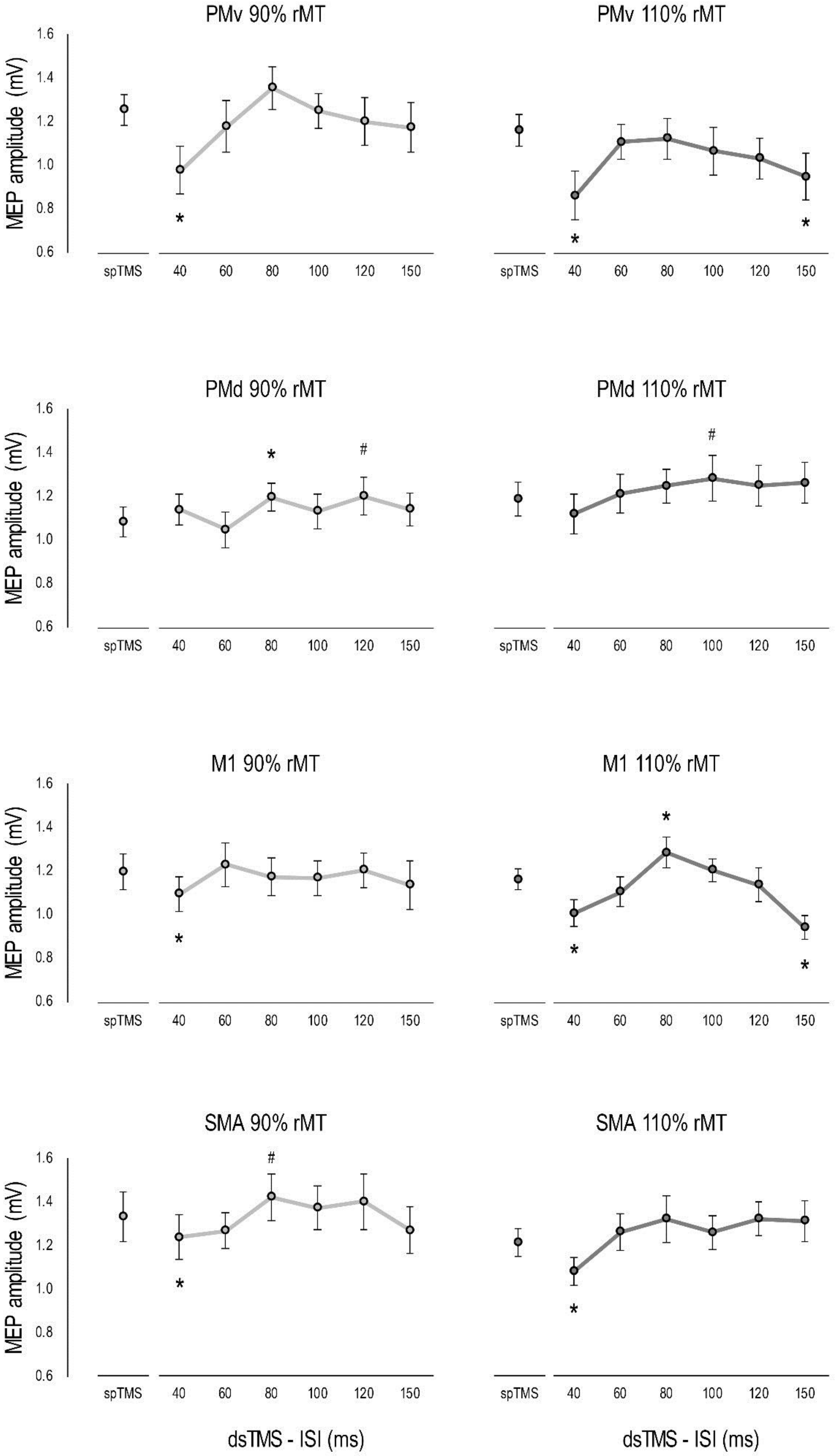

Supplementary Figure S1. Raw MEP amplitudes (in mV) induced by spTMS and dsTMS in each block (i.e., in each combination of CS site and CS intensity). Asterisks (*) indicate significant comparisons between dsTMS and spTMS MEPs ($P < .05$) and harsh marks (#) indicate non-significant trends ($.09 < P < .06$). In addition to the comparisons reported in the main text (ISI = 40, 80, 150; see Fig 1), here, further exploratory t-tests were conducted for those ISIs not associated with significant effects in the CS site x CS intensity ANOVAs (ISI = 60, 100, 120). These comparisons show sparse non-significant modulatory effects of premotor conditioning that did not emerge using our stringent criterion of site-specificity.
